# Tobacco-Free Duo Adult-Child Contract for Prevention of Tobacco Use Among Adolescents and Parents: Protocol for a Mixed-Design Evaluation

**DOI:** 10.2196/21100

**Published:** 2020-10-29

**Authors:** Maria Rosaria Galanti, Anni-Maria Pulkki-Brännström, Maria Nilsson

**Affiliations:** 1 Department of Global Public Health Karolinska Institutet Stockholm Sweden; 2 Centre for Epidemiology and Community Health Stockholm Sweden; 3 Department of Epidemiology and Global Health Umeå University Umeå Sweden

**Keywords:** tobacco use, prevention, school, social influence, public commitment, cluster randomized trial, observational study

## Abstract

**Background:**

Universal tobacco-prevention programs targeting youths usually involve significant adults, who are assumed to be important social influences. Commitment not to use tobacco, or to quit use, as a formal contract between an adolescent and a significant adult is a preventive model that has not been widely practiced or explored and has been formally evaluated even less. In this paper, we present the rationale and protocol for the evaluation of the Swedish Tobacco-free Duo program, a multicomponent school-based program the core of which rests on a formal agreement between an adolescent and an adult. The adolescent’s commitment mainly concerns avoiding the onset of any tobacco use while the adult commits to support the adolescent in staying tobacco free, being a role model by not using tobacco themselves.

**Objective:**

To assess (1) whether Tobacco-free Duo is superior to an education-only program in preventing smoking onset among adolescents and promoting cessation among their parents, (2) whether exposure to core components (adult-child agreement) entails more positive effects than exposure to other components, (3) the impact of the program on whole school tobacco use, (4) potential negative side effects, and (5) school-level factors related to fidelity of the program’s implementation.

**Methods:**

A mixed-design approach was developed. First, a cluster randomized controlled trial was designed with schools randomly assigned to either the comprehensive multicomponent program or its educational component only. Primary outcome at the adolescent level was identified as not having tried tobacco during the 3-year junior high school compulsory grades (12-15 years of age). An intention-to-treat cohort-wise approach and an as-treated approach complemented with a whole school repeated cross-sectional approach was devised as analytical methods of the trial data. Second, an observational study was added in order to compare smoking incidence in the schools participating in the experiment with that of a convenience sample of schools that were not part of the experimental study. Diverse secondary outcomes at both adolescent and adult levels were also included.

**Results:**

The study was approved by the Umeå Regional Ethics Review Board (registration number 2017/255-31) in 2017. Recruitment of schools started in fall 2017 and continued until June 2018. In total, 43 schools were recruited to the experimental study, and 16 schools were recruited to the observational study. Data collection started in the fall 2018, is ongoing, and is planned to be finished in spring 2021.

**Conclusions:**

Methodological, ethical, and practical implications of the evaluation protocol were discussed, especially the advantage of combining several sources of data, to triangulate the study questions. The results of these studies will help revise the agenda of this program as well as those of similar programs.

**Trial Registration:**

International Standard Randomized Controlled Trial Number (ISRCTN) 52858080; https://doi.org/10.1186/ISRCTN52858080

**International Registered Report Identifier (IRRID):**

DERR1-10.2196/21100

## Introduction

Social influences, such as smoking parents, siblings, or friends, are the strongest determinants of smoking initiation in adolescence [1,2]. In practice, one may conceptualize smoking initiation as a socially learned behavior, while the subsequent trajectories leading to escalation or to extinction of smoking behavior are determined by a more complex mixture of reinforcing or weaning factors, including genetics, psychosocial circumstances, and availability of tobacco products [3].

According to Bronfenbrenner’s theoretical frame [4], people in close microenvironments are likely to exert the strongest health-related influences (in this case pro or antismoking), at least in early adolescence and during initial episodes. Peers normally exert a direct and proximal influence by creating the context for smoking to occur; in fact, the association of friends or siblings smoking is in many cases stronger than that of parents smoking [5]. Adult influences, on the other end, are conveyed on a broader frame. First, adults may shape opinions and attitudes over developmental years by simply tolerating or not explicitly disapproving of smoking [6]. Smoking behavior among significant adults may further encourage the onset of smoking in adolescence by creating an implicitly favorable norm [7]. Availability of cigarettes with or without permission is high in environments where adults smoke [8], which is an important facilitating factor when purchase is not affordable as it is the case among adolescents, a price sensitive group [9]. Also, the possibility that early exposure to environmental smoking primes the child’s brain to the rewarding properties of nicotine has been presented [10].

Importantly, social influence seems to extend to tobacco uses other than smoking, at least in countries where other established forms of tobacco use exist, as is the case in Sweden, where the use of the oral moist tobacco snus is widespread among men [11].

For these reasons. adult influences (and parents’ influences, in particular) are usually regarded as an important cue in universal prevention of tobacco use.

Consistent with these observations, youth smoking prevention programs in different settings have often included some degree of parental involvement [12]. Despite promising indications that adding a family component increases the effectiveness of school-based programs, the findings of studies have not been consistent [13]. Some encouraging evidence exists on using public commitment as a strategy for prevention. In a systematic review [14] of school-based smoking prevention programs, there was a significant effect of programs including commitments to not smoke. A community-wide school-based intervention program aimed at preventing adolescent tobacco use, incorporating public commitment activities as one of several components, led to a reduced smoking in those who initiated smoking [15].

A component of public commitment has been successfully included in a variety of public health programs and target groups. For instance, in programs using life skills training to prevent drug use, some positive results have been found when including this component [16]. In a school-based diabetes prevention project, there was a suggestion that a public commitment to a healthy lifestyle was associated with a lower prevalence of obesity at follow-up [17]. Some support has also been found for interventions using public commitment as a strategy in adult smoking cessation, including the use of contracts in general practitioner-led interventions [18].

Concerning smoking prevention in youths, a comprehensive social influence program including a decision-making component was found to be effective in a randomized trial [19] for students aged 13-15 years. An evaluation of a prevention program against substance use and other problem behaviors among adolescents in grade 9 using a contract as a component reported small to moderate differences in substance use between groups based on length of participation in the prevention program; however, limitations in the study design made it difficult to draw firm conclusions [20]. A school-based smoking prevention program called the Smoke Free Class competition was introduced in more than 20 European countries during the years 1997-2009. The program included a commitment to not smoke and prize draws. The program was evaluated by randomized controlled trials in several countries, and a meta-analysis [21] reported that the program seemed to be effective in preventing smoking.

The Swedish program *Tobacco-free Duo* is an example of this category of programs, addressing all forms of tobacco use. Details on the intervention and on its preliminary evaluation have been published [22-24]; however, firm conclusions on favorable effects of the intervention could not be reached due to weak study design or to methodologic shortcomings in these earlier evaluations. The Public Health Agency of Sweden commissioned this evaluation in order to strengthen the evidence base to be incorporated in possible future recommendations.

This commission was translated into the following specific objectives: (1) to assess whether the comprehensive Tobacco-free Duo program is superior to an education-only program in preventing onset of use of cigarettes or other tobacco among adolescents or in fostering smoking cessation among smoking parents, (2) to assess whether the adolescents or parents exposed to the core component of the program (adult-child agreement) refrained from using tobacco to a higher extent than those who were only exposed to other components of the program, (3) to explore the impact of the program on the whole school tobacco use prevalence over time, (4) to understand whether receipt of the program entailed negative side effects on the participating adolescents, and (5) to analyze school-level factors related to fidelity of the program implementation.

A health economic evaluation (cost-effectiveness study) will be conducted alongside the randomized controlled trial, and its corresponding protocol will be the object of a separate publication.

## Methods

### Intervention

#### Overview

The Tobacco-free Duo is a complex intervention for the universal prevention of tobacco use developed in Sweden and in use since 1993, starting from the County of Västerbotten in the North of Sweden. From 2007, the intervention has also been adopted in other counties, by request of the regional government or by request of the individual municipalities or schools, therein. About 80 municipalities are known to have implemented the Tobacco-free Duo program [25].

#### Components

In brief, the program aims to mobilize antitobacco practices and attitudes in adolescents’ near environments, with schools as main promoters and arenas for the activities. The recommended starting point is in the sixth or seventh grade of compulsory school (between 12 and 13 years of age), and it encompasses 6 central components that were manualized in this study.

1. The tobacco-free pair (Duo) core component that is the origin of the program’s name consists of an agreement between an adolescent and a significant adult (at least 18 years old) elected as partner by the adolescents. The pair agrees to remain tobacco free during the following 3 years, at least, until the index adolescent leaves the compulsory school (about 15 years of age). The written agreement is signed by both partners, possibly during a public event in school, strengthening the mutual commitment. However, a pair can be formed anytime during the school year.
2. Student information is given by a member of the school staff who informs the student in the sixth or seventh grade about the school choice to adopt the program, briefly discusses tobacco control issues, and actively encourages participation of the adolescents.
3. Parent information is given during an ordinary parent meeting at school where they are given access to information and materials explaining their role in tobacco prevention and how they can actively support their children in their commitment.
4. As an incentive to participate, all adolescents signing a contract will receive a membership card that entitles them to some fringe benefits in local shops or leisure-time activities.
5. At the end of each school year, the pairs disclose and confirm their tobacco-free status. The disclosure entitles the index adolescent to participate in a lottery taking place just before the summer break.
6. Interactive classroom education is conducted by trained school personnel in all classes during grades 6 (or 7) to 9. This education consists of age-specific information and practical exercises (eg, how to identify and resist social influences). For the purpose of this evaluation, the educational part was further structured and manualized before training personnel.

### Effectiveness Evaluation

The evaluation protocol has been developed in order to address the objectives of the study through the following primary and secondary questions, formulated according to a PICO (population, intervention, comparator, outcome) framework [26]. It should be observed that, for feasibility purposes (due to educational block organizations in Swedish schools where many children change school between sixth and seventh grade), in this evaluation, the start of the intervention was set in the seventh grade (12- to 13-year old adolescents).

### Study Design

The evaluation questions will be addressed through a mixed-design approach, where experimental and nonexperimental designs will be combined.

#### Experimental Design

A parallel cluster randomized controlled experiment will be conducted with schools as units of randomization and individual students as unit of analysis. A superiority approach will be employed for hypothesis testing (ie, the null hypothesis to be rejected will be that the Tobacco-free Duo comprehensive intervention is either inferior or equivalent to the education-only component). Through this design, questions 1 to 5 will be addressed (Tables 1 and 2). The trial was registered (ISRCTN 52858080) on January 4, 2019 (ie, after enrollment of the first participant but prior to baseline assessments being completed).

**Table 1:** Evaluation questions.

Objective–question		Formal question definition	
**Objective 1**			
	Questions 1 and 3		Is the probability of having never smoked (question 1) or used any tobacco product^a^ (question 3) at the end of follow-up in the ninth grade higher for seventh-grade adolescents in schools assigned to the full program (Tobacco-free Duo components 1-6) compared to that of adolescents in schools assigned to conduct only the educational component (component 6)?
	Questions 2 and 4		Is the probability of never having progressed to regular (weekly during at least 3 consecutive months) use (question 2: smoking cigarettes; question 4: any tobacco product) by grade 9 higher for adolescents in Tobacco-free Duo schools than for those in education-only schools?
**Objective 2**			
	Question 5		Is the probability of having refrained from using cigarettes or any tobacco by the end of follow-up higher among adolescents and parents who smoke who signed the formal agreement to become a tobacco-free pair (core component) compared to those who only received other components?
	Question 6		Is the proportion of sustained quitters (no smoking in the past 30 days) among parents who smoke before the program’s start higher when the index child attended a Tobacco-free Duo school compared to an education-only school, at the end of the compulsory grades?
**Objective 3**			
	Question 7		Is the total prevalence of never smokers among students in the grades 7 to 9 declining more slowly over 3 years in Tobacco-free Duo schools compared to education-only schools or schools delivering their usual antismoking programs (external reference group schools)?
**Objective 4**			
	Question 8		Are there undesirable side effects of the full-component intervention, such as: exclusion or frequent dropout from child-adult contracts of adolescents in families of low socioeconomic status; or worsening of perceived mental or general health among participants receiving the Tobacco-free Duo intervention compared to those only receiving the educational component?

^a^The term *any tobacco product* encompasses the use of Swedish smokeless tobacco snus and of e-cigarettes.

**Table 2:** PICO questions addressed in the experimental (cluster randomized trial) and nonexperimental (observational) studies for the effectiveness evaluation of Tobacco-free Duo.

Question		PICO^a^ description	
**Experimental**			
	**Superiority of Tobacco-free Duo comprehensive intervention vs education only in preventing smoking/tobacco onset**		
		Population	Adolescents 13 years old at baseline with valid parental consent—average follow-up 38 months
		Intervention	Tobacco-free Duo 6 components
		Comparator	Educational component of Tobacco-free Duo
		Outcome 1	Never smoked (negative answer to the question: Did you ever try smoking, even a few puffs?)
		Outcome 2	Never smoked regularly (negative answer to the question: Did you ever smoke weekly for 3 or more months in a row?)
		Outcome 3	Never used any tobacco (negative answer to both questions: Did you ever try smoking, even a few puffs? Did you try smokeless tobacco?)
		Outcome 4	Never used any tobacco regularly (negative answer to both questions: Did you ever smoke weekly for 3 or more months in a row? Did you ever use snus weekly for 3 or more months in a row?)
	**Superiority of Tobacco-free Duo comprehensive intervention vs education only in determining smoking cessation among participants’ parents**		
		Population	Parents of adolescents participating in the trial who reported smoking at baseline
		Intervention	Signing an adult-child contract
		Comparator	Receiving other intervention components, no contract
		Outcome	No past 30-day smoking at follow-up
**Nonexperimental**			
	**Effect of adhering to the core component *child-adult contract***		
		Population 1	Adolescents participating the longitudinal assessment of the cluster randomized trial
		Intervention	Signing an adult-child contract (as treated)
		Comparator	Receiving other intervention components, no contract (as treated)
		Outcome	Never smoked or used tobacco at follow-up in ninth grade
		Population 2	Parents of adolescents participating the longitudinal assessment of the cluster randomized trial, baseline smokers
		Intervention	Signing an adult-child contract
		Comparator	Receiving other intervention components, no contract
		Outcome	No past 30-day smoking at follow-up
	**Decline in school prevalence of never smokers**		
		Population	Students registered in the grades 7-9 of the schools participating in the experimental study and in the schools serving as external reference during three consecutive years
		Intervention 1	Tobacco-free Duo 6 components (as treated)
		Intervention 2	Educational component of Tobacco-free Duo only (as treated)
		Comparator	Usual education or health promotion
		Outcome	Average point prevalence of never smoking in spring term of each school year

^a^PICO: population, intervention, comparator, outcome.

#### Nonexperimental (Observational) Design

Two different comparisons will be established. In the first comparison (addressing objective 2 question 6) an as-treated analysis of the trial data will be conducted where individual students assigned to the Tobacco-free Duo intervention and signing a child-adult contract will be contrasted to students receiving other Tobacco-free Duo components but not signing the contract. In the second comparison (addressing objective 3 question 7), all students attending schools in grades 7 to 9 in both experimental groups will be contrasted to students in a convenience sample of schools in the same broad catchment areas where the experiment takes place (an external reference group). Schools in this external reference group will be those willing to participate in the survey data collection but not in the experimental study. Therefore, they will represent the subset of schools in the target areas that will probably not be willing to adopt the program once released for dissemination. In Table 2, a summary is presented of the relevant comparisons established with an observational design.

### Intervention Implementation and Fidelity (Objective 5)

As a complement to the evaluation of effectiveness, we plan to monitor and describe the implementation of the Tobacco-free Duo intervention, in particular the average proportion of schools and classes delivering the intervention components as intended; the observed versus expected frequency of delivery of each of the 6 components and their range; and the characteristics of the schools not conducting or completing the program as intended and alleged reasons for this failure.

### Adolescents’ Experiences and Reported Side Effects of the Intervention (Objective 4)

We plan to identify negative outcomes of the intervention, particularly those connected with the administration of the core component agreement between an adult and a child. We will use the conceptual framework proposed by Lorenc and collaborators [27] to identify adverse effects in 2 domains: psychological side effects and equity side effects. Specifically, we will use adolescent questionnaire data to explore unequal distribution of application of the core components across socioeconomic status and composition of the index families (equity aspect). Reasons for not signing a contract or changing contract partner, satisfaction with the partnership, and perception of the partner’s support will also be explored (psychological aspects), both through questionnaire data and through in-depth interviews with adolescents. To this end, a convenience sample of both contract holders and nonholders will be enrolled.

### Study Procedures

#### Invitation and Selection of Schools

All schools located in 11 regions of central and south Sweden encompassing the higher block grades (7 to 9) will be invited to participate in the study through a letter addressed to the school’s headmaster at the end of the school year preceding the conduction of the intervention. We estimate this underlying source sample as about 600 schools. Schools will be eligible if they have at least 2 classes in the target grades and if they did not or do not plan to adopt the Tobacco-free Duo program until the evaluation is completed. The eligibility criteria will be assessed among respondents willing to participate through a questionnaire on school organization and characteristics sent concurrently with the invitation.

The final selection will rest on a formal agreement issued by the school headmaster to be randomly assigned to the intervention or comparator condition, to engage the school personnel in the training for and in the delivery of the intervention, and to facilitate the data collection. We estimate that at least 40 schools will be recruited into the experimental study.

Schools not willing to be randomized will be asked if they are willing to participate as part of the external reference group (ie, to deliver and report on the usual antismoking program if any and to collect data from students in repeated anonymous cross-sectional surveys).

#### Randomization

After recruitment, the consenting schools will be simultaneously randomly assigned to either the full program (Tobacco-free Duo) or to the education-only component of the same program. The random assignment will be performed by a statistician based at the steering group through a computer-generated series of random numbers, paired to each school, after stratification into publicly and privately run schools.

The results of the randomization will not be disclosed to the participating schools until the beginning of the school year during which the intervention will be delivered. Because of the nature of the interventions, blinding of participants will not be possible.

#### Identification and Enrollment in the Trial of Individual Students

Based on the school rosters, all students attending the seventh grade in the beginning of the school year will potentially be eligible for participation in the study. Besides the information delivered in class and school (see components 2 and 3), an individual invitation letter is sent to students’ home addresses, and individual parental consent will be sought with an opt-in procedure. In the invitation, it will be underlined that it will be possible to withdraw the assumed consent any time, both from the parents’ side and from the student’s side. Also, it will be clarified that the consent to participation concerns the baseline and follow-up collection of information to be entered in a register with personal identifiers but not the receipt of the intervention. The latter is decided at the school level, therefore not submitted to any individual consent, similar to any school-based activity.

Based on previous experience, we foresee that we will be able to enroll approximately 85% of the eligible students in the seventh grade. All recruitment procedures will be conducted by an executive team composed of research officers under the guidance of the principal investigators.

#### Training of School Personnel

School staff involved in the study are offered an annual meeting with education, training, and networking in their respective experimental group (Tobacco-free Duo school or education-only school). The content of the meeting is diversified according to the assigned intervention, with a common part regarding instructions for data collection and the educational intervention component. The aim is to gradually increase the personnel’s knowledge, to give an opportunity for exchange of experiences during the project period, and assure quality of data collection over time. The meetings are scheduled to be held annually before the fall term start, separately for education-only and Tobacco-free Duo schools.

#### Delivery of the Interventions

The school leadership at each participating school nominates a contact person and constitutes an operative team responsible for the implementation of the assigned intervention. The contact person will be responsible for contacts with the research group and for convening the school’s operative team. This includes the contact person, one or more persons from the student health care team, and one or more teachers in grades 7-9. Together they are responsible for the implementation of the assigned intervention components.

### Data Collection and Management

At both baseline and follow-up, information on outcome and predictor variables will be collected at 2 levels.

School-level information will encompass compositional and organization measures such as number of registered students and staff, staff average qualification, proportion of students with parents with lower than high school education, proportion of students with parents born outside Sweden, public or private management, and prior and current health promotion initiatives or preventive programs. Structured checklists and questionnaire forms will be developed.

In schools participating in the experimental part of the study, information will be continuously collected about the actual implementation of the intervention components and staff effort dedicated to them. To this end, a structured web-based form will be used.

Individual-level information will be gathered from individual students in Tobacco-free Duo schools, education-only schools, and reference group schools; from parents of students in Tobacco-free Duo and education-only schools; and adults in Tobacco-free Duo schools signing the contract with individual students, when not coinciding with one parent. All information will be collected with self-completed structured questionnaires with different content and administered with different procedures as shown in Table 3. All questionnaires will be based on sets of questions that have been previously used in Swedish surveys or longitudinal studies of tobacco use in youths. Individual participants in the experimental study will be traced through school records, even in cases of change of school or residence address. In fact, during compulsory education, changes of schools are registered at both ends of the transition (former and new school). Those absent during data collection days will be reached at their latest registered residence addresses.

**Table 3:** Data collection instruments, information, and procedures.

Population	Instrument and main domains	Administration	Identifier	Time-points
Participating students in schools randomized to Tobacco-free Duo or education only	Paper questionnaire, with information on: Cigarette and snus use (any, current, frequency, quitting) Tobacco use among friends and family members Received education, knowledge and attitudes toward tobacco Recent and current use of alcohol Ever use of illicit drugs General health, physical activity, and sedentary time Perceptions about the contract (if appropriate)	Self-administered in the classroom	Unique study code matched to identity information	Baseline: fall term of school year 1- grade 7 Follow-up 1: end of school year, grade 7 Follow-up 2: end of school year 2, grade 8 Follow-up 3: end of school year 3. grade 9
Guardians of the participating students above	Paper questionnaire, with information on: Sociodemographics Relation with the index child and perception of risks with tobacco use Own tobacco and alcohol use	Self-administered at home	Same code as the index child above	Same time points as above
Adults in Tobacco-free Duo pairs	Paper questionnaire, with information on: Own use of cigarette and snus Perception of own role in the intervention	Self-administered at home	Same code as the index child above	Anytime a contract is signed
All students registered in Tobacco-free Duo schools, education-only schools and reference group schools in grades 7-9	Paper questionnaire, with information on: Ever, regular and recent use of cigarette and snus	Self-administered in the classroom	No identifier (anonymous survey)	Cross-sectional surveys at the end of school year 1, 2 and 3

**Figure 1:**
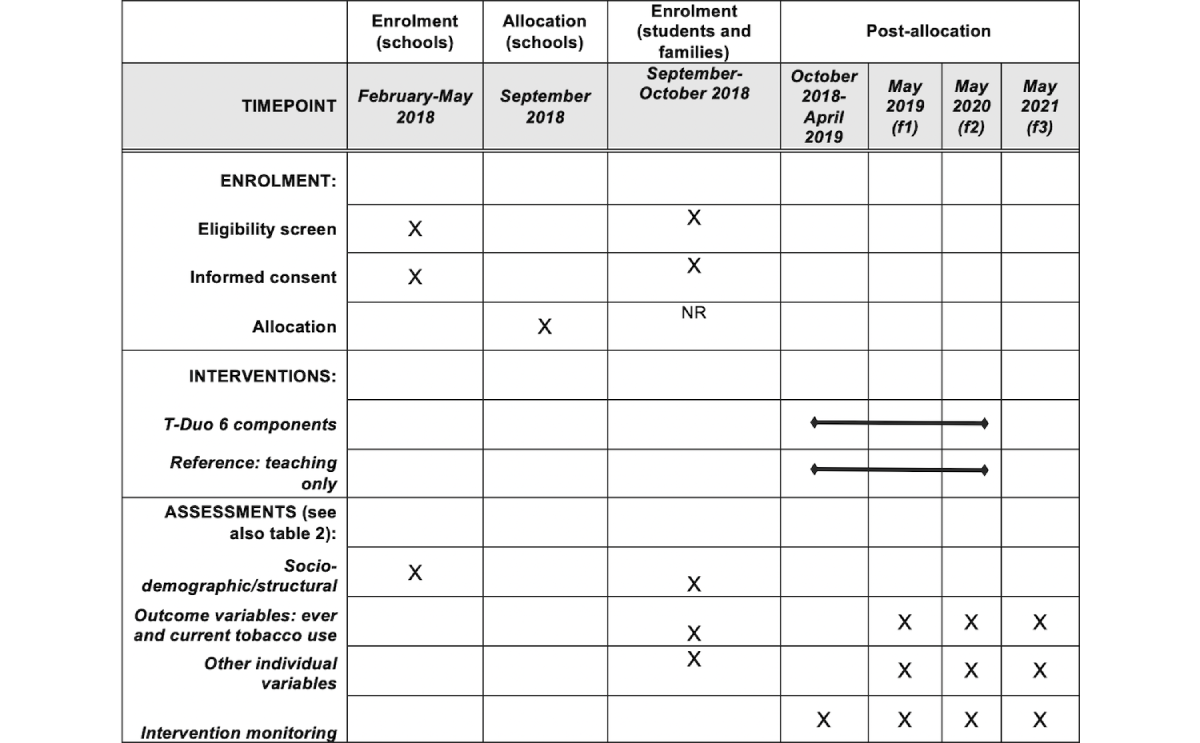
Schedule of enrollment, interventions, and assessments related to the experimental study (cluster randomized trial).

Figure 1 displays a scheme of the participants’ enrollment and assessments time-points for the experimental part of the study.

Individual data from questionnaires will be optically scanned, and the corresponding electronic files will be stored in a password-protected section of the server at the Department of Global Public Health, Karolinska Institutet. In order to preserve strict confidentiality, this data will be stored without personal identifiers, substituted by a study code unique to each participant. Personal identifiers will be kept in a separate section of the same server, only accessible to 2 members of the study team, responsible for data linkage and follow-up procedures, respectively. Ten years after the study start the personal identifiers will be removed and all information will be kept identified only through the study code.

### Statistical Methods

#### Estimation of Sample Size for the Experimental Study

We estimated that an optimal sample of 3000 students (about 40 schools) should be recruited in order to detect as statistically significant at the 5% level a risk ratio intervention to control of 1.10 based on the primary outcome (never smoker in the ninth grade). The estimation is based on the following assumptions: desired power 80% or higher; design effect due to the cluster design 1.98, an average cluster size of 50, and an intraclass correlation coefficient of 0.02; prevalence of the outcome in the minimal intervention (control) group 0.70; eligible students at baseline (never smoke) 92%; attrition between seventh and ninth grade 20%; and ratio intervention to control 1:1.

#### Data Analysis

Data from the trial will be analyzed primarily according to an intention-to-treat approach, preserving the random assignment [28]. Missing outcome information because of attrition at follow-up will be addressed by means of sensitivity analyses including last observation carried forward; best- and worst-case scenario, assuming missing answers as representing either the best or the worst outcome; and multiple imputation if a missing at random assumption for the missing data will not be dismissed [29].

Secondary analyses will be conducted per protocol, thus including only students in schools that adhered to at least 80% of the whole assigned intervention. Furthermore, an as-treated analysis will be conducted, comparing the outcome among students exposed to different components and intensities of the intervention, in particular the adoption of the core component *child-adult agreement* (research question 6). These approaches disregard randomization, therefore they are prone to producing biased estimates of the effect unless adjusted for most of the potential confounders [30]. However, they may be useful in order to assess the consistency between an expected effect and the intervention’s theoretical rationale [30] and in order to gain insights on the reasons for not detecting the estimated effect [31].

Finally, we will be able to compare the prevalence trends across 3 school years as well as the final prevalence at the end of follow-up between schools participating in the experimental study and schools in the external reference group, carrying on usual health education. This will enable us to estimate the potential impact of the intervention when released.

### Ethics and Dissemination

#### Ethics Approval and Consent to Participate

The study was approved by the Regional Ethics Review Board, Umeå (registration number 2017/255-31). Participant schools and individuals were required to give explicit informed consent to data collection, analysis, and reporting prior to inclusion in the study. Written and verbal operator-recorded consent has been obtained from parents or guardians of participating minors. The procedure approved by the Ethic Review Board included verbal and even opt-out consent.

#### Coordination of the Study, Roles, and Responsibilities

The several study components described in this protocol will be jointly coordinated by Umeå University (principal investigator: MN) and Karolinska Institutet (principal investigator: MRG). A steering group based at Umeå University will be responsible of all decisions concerning the scientific integrity of the protocol, amendments to the protocol, and the consequent operative procedures, including data monitoring and the decision to terminate the study. Besides the principal investigators, the group will include a researcher in charge of the economic evaluation, a statistician and data manager, and a senior research officer coordinating the field activities.

Twice a year, the steering group will report on the conduct of the trial to the funder (Public Health Agency of Sweden), and will agree on a plan for the dissemination of the results of the study besides the freedom of scientific publication in peer-reviewed journals. The dissemination plan will include interim news on the funder’s website, newsletters to participant schools and students, public lectures and educational occasions, and press-releases in relevant cases.

A data share policy will be also developed and maintained by the steering committee. This usually entails a formal written agreement with the requesting investigators and agencies to commit themselves to the same confidentiality levels as the leader institution.

## Results

Recruitment of schools started in the fall of 2017 and continued until June 2018. In total, 43 schools were recruited to the experimental study, and 16 schools were recruited to the observational study. Data collection started in the fall 2018 and is ongoing. The last round of data collection is planned for the spring 2021. Data analysis of baseline characteristics is due to commence, and first results are expected at the end of 2020.

## Discussion

This study protocol, for the evaluation of a complex intervention for the universal prevention of tobacco use initiation among early adolescents, proposes a mixed methods approach, combining the reciprocal strengths of a randomized experiment and of observational studies, as well as primary and secondary analyses of both study designs.

The protocol has several strengths, compared to similar studies. First, it aligns with the current increasing consensus on the importance of mixed methods [32] and of triangulation approaches in accruing robust evidence in the evaluation of interventions [33-35]. Two aspects of community trials call for triangulation approaches. On the one side, experiments establish the effect of being randomized to specific conditions, which in pragmatic trials do not coincide necessarily with the receipt of the intervention [31]. By conducting an as-treated analysis [30] according to the actual implementation of and adherence to the intervention, we aim to triangulate the question of effectiveness of Tobacco-free Duo program. By comparing groups no longer randomly assigned to the levels of the intervention, this analysis will have to take bias from confounding into account.

On the other side, participants in an experiment (be they organizations or individuals) constitute an imperfect representation of the underlying populations on which the inference should be drawn, because of the numerous selection steps involved in the conduction of an experiment [36]. In this study, we plan to include a comparison group that comprises schools that agreed to participate in annual surveys among all students in the 3 final compulsory grades but did not agree to be randomized to either experimental group. These schools are likely to well-represent the majority of Swedish schools (ie, the subgroup consisting of those that would not adopt the intervention if offered it at the scaling-up stage). By comparing the development over time of tobacco use in these schools with that of schools randomized to the minimal or comprehensive intervention (across a set of school-level characteristics), we aim to infer the added effect of the intervention on decline in tobacco uptake. For instance, if the prevalence of lifetime smoking or tobacco use similarly declines in reference group schools as in the randomized ones, we may project that the impact of a large and intensive dissemination of the intervention (even if effective in the randomized comparison) would be minimal.

Furthermore, we propose the use of qualitative methods and of process data in order to achieve 2 additional objectives. The first objective to be pursued is a deeper understanding of how the conduction of the intervention may differ between school organization characteristics. This knowledge is important in order to judge cluster-level confounding in nonrandomized studies [37]. An additional advantage of such a piece of knowledge would be the possibility to make projections of the potential benefits deriving from the application of the method to large communities with a known distribution of the studied characteristics.

The second objective is to study the occurrence and the nature of undesirable side effects connected with the intervention when delivered as intended. While it is recognized that such effects are of importance in the study of medical or psychological treatments, much less attention has been devoted to methods for studying them in the domain of preventive interventions. One of the reasons behind this failure is the need to carefully conceptualize expected adverse outcomes in a logic framework (one cannot study all kind of potential outcomes). According to the framework presented by Lorenc and collaborators [27], we will focus on potential adverse effects connected with the core component of the intervention (ie, the adult-child agreement) in terms of psychological and equity adverse effects. Children not able to be supported by an adult may be particularly vulnerable for reasons that may or may not be connected to the target behavior. The publicity around the contract among peers and the presence of fringe benefits for those complying may increase the segregation of these psychosocial risks. Also, children whose adult partners “infringe” the agreement by starting or relapsing into smoking may experience a profound disappointment and loss of trust that undermines the relation with a significant other.

The third strength of this study protocol rests in the choice of a standardized comparison group in the randomized controlled trial design. Several community trials employ comparator conditions that are largely opportunistic (ie, rely upon the concept of usual conditions). While undoubtedly pragmatic, this approach may be misleading at the stage of judging on causal effects deriving from the application of an intervention that is the explicit scope of a randomized controlled trial. In fact, while the alternative intervention under investigation is often quite standardized, usual conditions are not, even if in a certain context (for instance, in a given school system), there may be some recurrent features. To clump together different conditions without any knowledge of their potential effects or even of their content may lead to biased results in any direction [38]. It is purported that the advantage of usual conditions would be to represent the background reality of which the actual participants would be a valid representation [37]. Anyone who has conducted experimental studies is aware that this is far from being true, rather the opposite. Participants in studies (be they experimental or not) are usually a nonrepresentative sample of the background populations (individuals or organizations), both because of the application of explicit inclusion and exclusion criteria and because of self-selection.

When the Tobacco-free Duo program started in the early 1990s, there was a limited amount of research on decision support and public commitments as components of public health interventions; the latter was mainly investigated among adults [18]. Since then, there have been some advancements in knowledge regarding intervention models including decision support and public commitment among young people, but this knowledge must still be regarded as insufficient. The proposed study will, therefore, add substantially to the empirical evidence in this regard.

## Abbreviations

PICO: population, intervention, comparator, outcome
